# A Novel Design to Enhance the Mechanical Properties in Cu-Bearing Antibacterial Stainless Steel

**DOI:** 10.3390/ma13020403

**Published:** 2020-01-15

**Authors:** Shaoheng Sun, Zhiyong Xue, Licong An, Xiaohua Chen, Yifei Liu

**Affiliations:** 1 Institute of Advanced Materials, North China Electric Power University, Beijing 102206, China; 2 Materials Engineering, Purdue University, West Lafayette, IN 47907, USA; 3 State Key Laboratory for Advanced Metals and Materials, University of Science and Technology Beijing, Beijing 100083, China; 4 Collaborative Innovation Center of steel Technology, University of Science and Technology Beijing, Beijing 100083, China

**Keywords:** copper precipitation, bimodal precipitations, hardness, ferritic antibacterial stainless steel

## Abstract

A novel method based on nano-scale precipitation hardening has been studied to strengthen copper-bearing ferrite antibacterial stainless steel. Bimodal precipitations can be observed after antibacterial annealing and low temperature aging treatment, which are large rod-shaped precipitates and nano-sized spherical precipitates, respectively. Due to two different morphological precipitates, the strength of the material is significantly improved without sacrificing formability, and at the same time, the excellent antibacterial properties remain. Under low temperature aging treatment, there is no obvious evidence to show the segregation at the interface between the rod-shaped copper precipitation and the matrix due to the low segregation coefficient of copper. The nano-sized copper precipitation uniformly nucleated and distributed on the matrix. The optimized heat treatment process is antibacterial annealing at 800 °C for half an hour followed by one-hour-aging treatment at 550 °C.

## 1. Introduction

With the improvement of living standards, antibacterial materials have become more and more popular nowadays for reducing the risk of bacterial infection [1]. More and more attention has been paid to antibacterial stainless steel because of its dual characteristics of structural material and antibacterial functional material [2,3]. Copper-bearing ferrite stainless steel has developed rapidly in recent years due to its low cost and good formability [4,5,6]. The excellent antibacterial property relies mainly on Cu^2+^ sterilization generated during the electrochemical reaction process [7,8]. The average daily release of Cu measured in Cu-bearing antibacterial stainless steel is much smaller than the daily recommended amount for the human body [9]. Low concentration of Cu^2+^ under a critical value is nontoxic and can improve the bone density of the human body [10,11]. However, only if the copper element precipitates out as copper precipitation and grows to a certain size can the copper precipitation kill the bacteria. When the copper element dissolved in the matrix or the size of copper precipitation is too small, the material cannot receive the antibacterial function [12,13,14]. So far, most copper-bearing ferritic antibacterial stainless steels are annealed at 800 °C to obtain rod-shaped copper precipitation with lengths of about 500 nm and widths of about 50 nm. Though the large copper precipitation gives the material excellent antibacterial properties, it cannot increase the strength of the material. The copper precipitation that plays the role of precipitation strengthening is very small and uniformly dispersed. Additionally, the nano-sized copper precipitation can be obtained by aging at low temperature [15]. In Cu-bearing pipeline steels, high-density fine copper nanoprecipitation could be obtained after aging at 500 °C for 1 h [16]. The hardness and strength are significantly improved when the copper-bearing steel is aged at about 500–550 °C for several hours [17]. After aging at 550 °C for 1.5 h, the yield strength and tensile strength, respectively, are increased by 270 MPa (147%) and 200 MPa (54%) in the 1.18 wt.% Cu-bearing interstitial-free steel (IF) steel [18]. The nano-sized copper precipitation contributes to strength and work hardening rate, which can be explained by the Orowan hardening mechanism [19]. Hui Guo et al. have indicated that after tempering at 600 °C for 18 h, the hardness of 1.76 wt.% copper-bearing steel is significantly improved due to the presence of copper particles with the average diameter of 21.0 ± 6.0 nm [20]. In addition to the effects of copper precipitates on mechanical strength, copper precipitates reduce the ductile-brittle transition temperature without significantly reducing the elongation of the material, and optimize the impact toughness of the material by increasing the screw dislocation mobility at low temperatures [21]. Therefore, by precipitating nano-scale copper precipitation in low-carbon copper-bearing steel, an excellent overall mechanical property with high yield strength (700 MPa) and high elongation (greater than 16%) can be obtained [22]. Jun-Yun Kang has studied the influence of copper content and indicated that the hardness and the impact toughness could be optimized at around 1 wt.% of Cu addition [23]. Since the antibacterial annealing temperature of copper-bearing ferritic stainless steel is higher than its aging strengthening temperature, the solid solution of copper in the matrix is still in a supersaturated state during the aging treatment. The key to uniformly disperse the copper precipitation as the second phase is the uniform formation of the nucleation. In copper-bearing niobium-microalloyed high strength steels, the nucleation positions of copper precipitation are different during the thermo-mechanical processing and isothermal aging process. During thermomechanical processes, copper precipitation nucleates at the polygonal ferrite or the interface between austenite and ferrite. During isothermal aging, however, copper precipitation is formed at the dislocations [21]. The dislocation density of Cu-bearing ferritic stainless steel is very low after a long time of high temperature antibacterial annealing, and a large amount of rod-like copper precipitation occurs at the same time. In the subsequent low temperature aging process, nano-scale copper precipitation either nucleates on the interface between the substrate and rod-shaped copper precipitates or uniformly forms on the substrate. The nucleated nano-scale copper precipitation can determine whether the strength of the material increases or not.

Thermo-Calc is always used to calculate the equilibrium thermodynamics in steel [24,25,26]. A description of the amounts of copper precipitation as a function of temperature can be obtained.

In the present research, the feasibility of dispersion strengthening of nano-sized copper precipitation will be discussed in Cu-bearing ferritic stainless steel based upon Thermo-Calc calculation. Additionally, the effect of nano-scale copper precipitation on microstructure, hardness and formability will be studied.

## 2. Materials and Experiments

**Figure 1:**
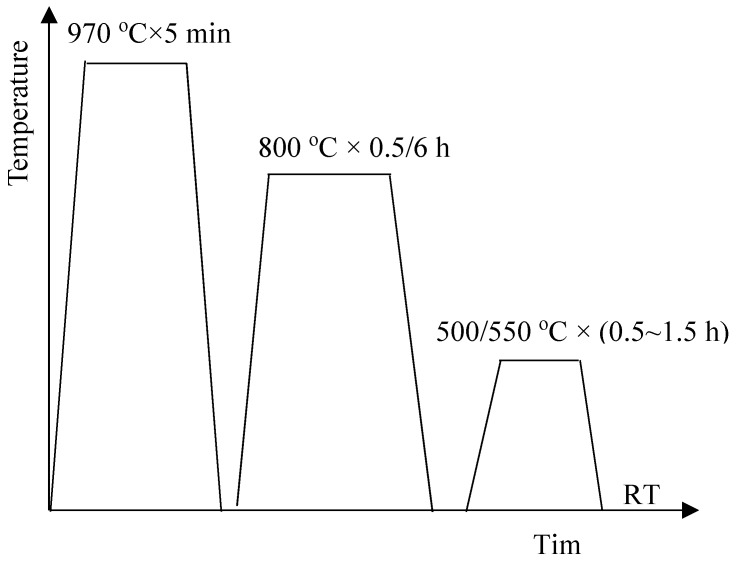
Schematic diagram of heat treatment, where RT refers to room temperature.

The tested steel in the current study was provided by Taiyuan Iron and Steel Corporation. The chemical composition of the tested steel is given in Table 1. The tested steel was hot-rolled to 4 mm thick after being forged, and then the slabs were cold-rolled to 1 mm thickness. After solution annealing at 970 °C for five minutes, the antibacterial annealing was performed at the temperature of 800 °C in this paper. Time of the antibacterial annealing is 0.5 h or 6 h. Aging at low temperature followed. Time of the aging is 0.5~1.5 h. The heat treatment schematic diagram is shown in Figure 1. When annealed at 970 °C, the copper element also dissolved Cu in the ferrite matrix. When annealed at 800 °C, the rod-shaped copper precipitation would be obtained, which can give the material excellent antibacterial properties. When annealed at 500/550 °C, the nano-sized copper precipitation would be obtained, which can improve the strength of material.

The copper precipitation was characterized by transmission electron microscopy (TEM), FEI Tecnai F20 (Hillsboro, OR, USA). Size of the specimen is *φ*3 mm × (<50 μm). The sample number of each process is three. The samples were prepared by double-jet thinning in the electrolyte of 5% perchloric acid and 95% glacial acetic acid. Specimens for hardness test were first ground with 1500-grit silicon carbide paper and then polished with 0.1 μm diamond paste. The hardness test was performed by Vickers microhardness tester (MVK-H21, Shanghai, China). The load was 500 g and the loading time was 10 s. Specimen size was 10 mm (rolling direction) × 8 mm × 1 mm. Number of tests was 5. The tensile specimens with 1.0 mm in thickness were cut from the sheet parallel to the rolling direction by a wire-electrode machine. The gauge length was 25 mm. After removal of oil and oxide skin, the samples were tested on a SANS XYB605C universal testing machine (MTS Corporation, Eden Prairie, MN, USA) at a speed of 1 mm/min. Three times for each condition were performed. According to GB/T4156-2007, the size of the sample for formability test was 70 mm × 70 mm × 1 mm. The test was performed on sheet forming machine, Zwick-BUP600(Ennepeta, Germany). Three samples were prepared to average the Erichsen index (EI) value. All samples were cut from the middle of the sheet.

According to JIS Z 2801:2012, the antibacterial tests were performed by Test Center of Antibacterial Materials, Technical Institute of Physics and Chemistry, Chinese Academy of Sciences, Beijing. The size of the antibacterial sample was 50 mm × 50 mm × 1 mm. Three samples were prepared to take the average of the antibacterial rate. The type of bacteria was *E. coli*. (ATCC 25922). The tested samples were sterilized by boiling at 121 °C for 20 min. 0.4 mL of the culturing solution containing the bacteria with (2.5–10) × 10^5^ cfu/mL was added on the tested steel. After incubating on the samples, the culturing solution was incubated for 24 h at 35 ± 1 °C on the nutrition agar plate.

## 3. Results and Discussion

### 3.1. Microstructure

**Figure 2:**
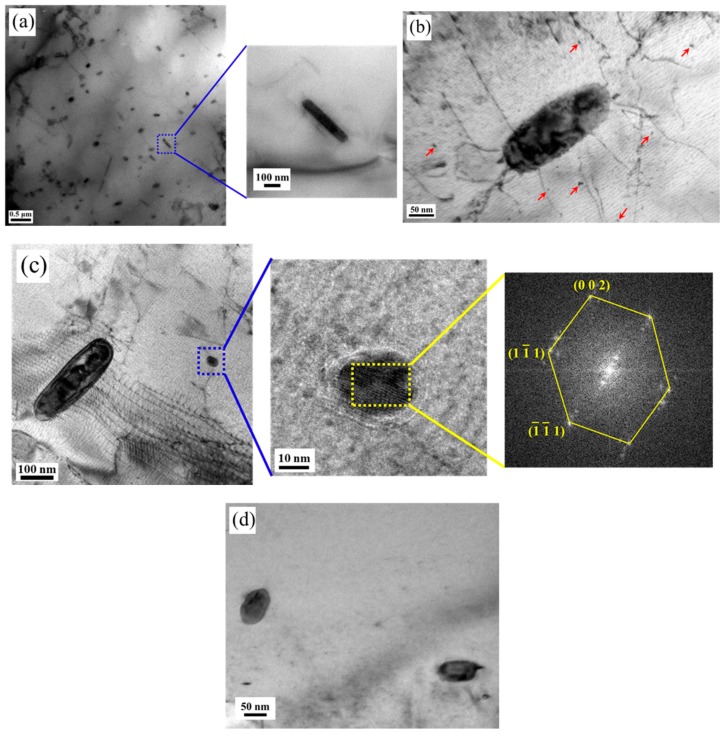
Microstructure of the copper precipitation before and after aging at 550 °C. (**a**) 0 h, (**b**) 0.5 h, (**c**) 1.0 h, (**d**) 1.5 h.

The morphology and microstructure of copper precipitation before and after aging at 550 °C are shown in Figure 2. Figure 2a shows the microstructure before aging, where only rod-shaped copper precipitations with length of about 400 nm and width of about 80 nm appeared. There is no fine copper precipitation on the matrix. Microstructures of copper precipitation after aging at 550 °C for half an hour are shown in Figure 2b. In addition to the huge rod-shaped copper precipitation, there are also some very fine spherical copper precipitations on the matrix. Some fine spherical copper precipitations with the size of about 5 nm appeared on the substrate. Bimodal precipitations are obtained, namely the rod-shaped copper precipitation and nano-sized copper precipitation. As the aging time increases, the fine copper precipitations continue to grow. When the aging time extends to one hour, the size of the fine copper precipitations reaches about 20 nm, as shown in Figure 2c. From the FFT image of the region in the yellow square box, the structure of the nano-sized copper precipitation is FCC, and a stress ring can be observed around the fine copper precipitate. This is because the atomic radius of iron and chromium are smaller than the atomic radius of copper, and the copper precipitations have the FCC structure, which is different from the BCC structure of the matrix, so the atomic arrangement at the interface is distorted. As shown in Figure 2d, when the aging time is extended to 1.5 h, the size of fine copper precipitation increases to 50 nm.

### 3.2. Antibacterial Test

**Figure 3:**
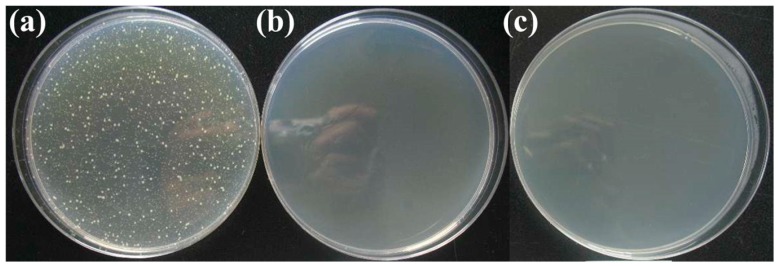
The photos of antibacterial effect against *E. coli.* (**a**) negative sample; (**b**) sample before aging; (**c**) sample after aging.

The photos of antibacterial effect against *E. coli.* are shown in Figure 3. Figure 3a is the negative comparison sample, where a large amount of bacteria is shown. Figure 3b,c are antibacterial results before and after low temperature aging, respectively. Few bacteria can be seen on both of the two samples, which indicate that low temperature aging will not reduce the antibacterial properties of the material.

### 3.3. Hardness

**Figure 4:**
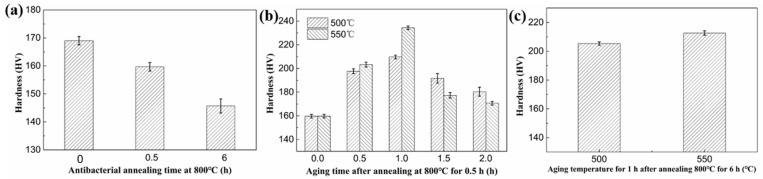
Hardness change of antibacterial stainless steel with (**a**) antibacterial annealing time at 800 °C; (**b**) aging time after annealing at 800 °C for 0.5 h; (**c**) aging temperature for 1 h after annealing at 800 °C for 6 h.

The hardness change of the antibacterial stainless steel of different heat treatments is shown in Figure 4. When 2.0% copper is completely dissolved in the matrix before annealing, the hardness of the tested steel is 169 ± 1.5 HV. After antibacterial annealing at 800 °C for 0.5 h and 6 h, the hardness of material decreases to 159.7 ± 1.5 HV and 145.7 ± 2.5 HV, respectively, as shown in Figure 4a. The decrease in the hardness of the material is due to the high antibacterial annealing temperature, and the size of the rod-shaped copper precipitates is particularly large, which has no precipitation strengthening effect on the steel. On the other hand, due to the added copper atom, the local non-uniformity in the lattice makes plastic deformation more difficult by impeding dislocation motion. Hence, the hardness can be improved. The effect of solid solution strengthening is proportional to the content of copper element. The content of Cu dissolved in the ferrite matrix decreases because of the rod-shaped copper precipitation, reducing the effects of solid solution strengthening, and thus, the hardness decreases. After antibacterial annealing for 0.5 h, a low temperature aging treatment is performed. When aged at 500 °C and 550 °C, the hardness of the tested steel both increases first and then decreases with time as shown in Figure 4b, which is because of the nucleation of nano-copper precipitation during aging treatment. As the aging time prolongs, the nano-sized copper precipitation gradually grows up and the hardness of the material increases. When the aging time is further increased, the over-aging occurs, which results in the formation of large-size copper precipitation, and thus, the hardness is reduced. The optimum aging time is one hour, whether aged at 500 °C or 550 °C. Under these conditions, the hardness values of the samples both reach peaks, namely 209.7 ± 1.5 HV and 234.3 ± 1.5 HV. In addition to the aging process, the antibacterial annealing time also has a great influence on the hardness. When the antibacterial annealing time is extended to 6 h, the hardness of the sample aged at 500 °C and 550 °C for 1 h decreases to 205.3 ± 1.2 HV and 212.7 ± 1.5 HV from 209.7 ± 1.5 HV and 234.3 ± 1.5 HV, respectively, as shown in Figure 4c. It can be seen that the strengthening effect of aging treatment will be weakened over the antibacterial annealing time. This is because the size of rod-shaped copper precipitates increases over antibacterial annealing time, along with the reduction of copper content in the matrix, resulting in decreasing of the hardness of the samples. The hardness changing above indicates that the mechanical properties of antibacterial stainless steel can be improved by aging treatment. The optimized heat treatment process is antibacterial annealing at 800 °C for half an hour followed by aging at 550 °C for one hour.

**Figure 5:**
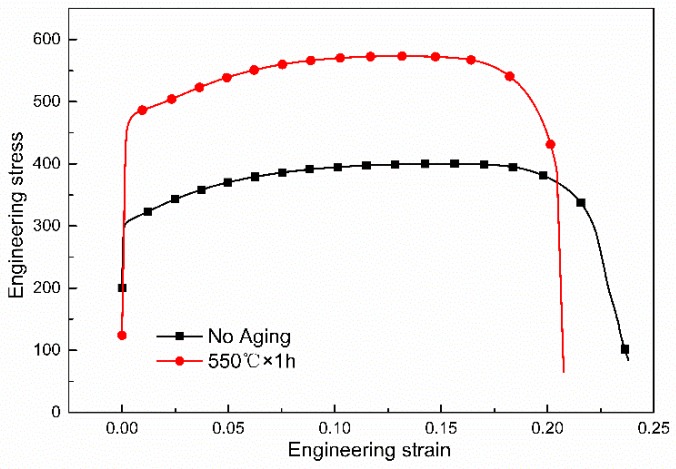
Engineering stress vs. engineering strain curves of the tested steels.

Figure 5 shows the engineering stress–strain curves of the tested steel and the mechanical properties are listed in Table 2. The conventional production process for Cu-bearing ferritic antibacterial stainless steel is annealing at 800 °C for 6 h to obtain big enough rod-shaped copper precipitation and meet the antibacterial property. The yield strength and tensile strength of the conventional sample with no aging are 309 ± 3 MPa and 403 ± 2 MPa, respectively. While the novel process in current study is annealing at 800 °C for 0.5 h and then aging at 550 °C for 1 h, the yield strength and tensile strength of the novel process with aging are 472 ± 3 MPa and 577 ± 4 MPa, respectively. The increase in yield strength is 163 MPa (52.75%) and that in tensile strength is 174 MPa (43.18%). The strength of a material is dependent on how easily dislocations can move. The nano-sized particles impede the movement of dislocations. Hence, the mechanical properties can be improved. The total elongation is reduced from 27 ± 1.0% to 22 ± 1.2%. The strengths are obviously improved and the total elongation is only slightly reduced. Yin has demonstrated that the yield strength and tensile strength are about 350 MPa and 490 MPa, respectively, when the antibacterial annealing is between 1 to 6 h in Fe-21.2Cr-1.52Cu-0.16Nb-0.13Ti-0.31Ni alloy [27,28]. Lin et al. have indicated that the yield strength and tensile strength are about 350 MPa and 480 MPa, respectively, when the material is under the process of hot rolling +850 °C × 10 h + cold rolling + 880 °C × 1 min in Fe-17.46Cr-1.44Cu-0.027C-0.023N alloy [29]. Though aging is performed in some studies, the yield strength and tensile strength are about 320 MPa and 515 MPa, respectively, when the material is under the process of 860 °C × 1 h + 550 °C × 4 h in Fe-17.15Cr-1.50Cu-0.023C-0.025N alloy [30]. The strengths in above studies are lower than that of this paper in novel process.

### 3.4. Formability

**Figure 6:**
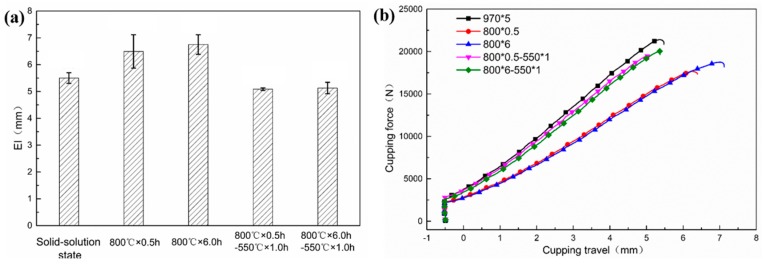
Formability under different processes (**a**) EI value; (**b**) cupping force–travel curves.

Figure 6 shows the relationship between formability and heat treatment. The Erichsen index (EI) is always used to describe formability property, and higher EI value indicates better formability [31]. The EI values of tested steel with different heat treatment are shown in Figure 6a. The EI value of the sample after solid solution treatment is 5.50 ± 0.20 mm. With the subsequent antibacterial annealing treatment, the EI values begin to increase. The EI value increases from 6.49 ± 0.62 mm to 6.75 ± 0.37 mm as the annealing time grows from 0.5 h to 6 h. However, EI value decreases as a consequence of low temperature aging treatment. The EI value of the sample with process of 800 °C × 0.5 h–550 °C × 1 h is 5.09 ± 0.05 mm, and that of the sample with process of 800 °C × 0.5 h–550 °C × 1 h is 5.13 ± 0.21 mm. The cupping force–travel curves are plotted in Figure 6b, where the cupping force is inversely proportional to the cupping travel. The comprehensive analysis of Figure 4 and Figure 5 shows that the higher strength, the worse formability. However, no significant change in formability behavior can be observed aspired by the stable Erichsen index, which also demonstrates that the nano-sized copper precipitation can obviously improve the strengths and plasticity is only slightly reduced.

### 3.5. Solid Solubility of Copper

**Figure 7:**
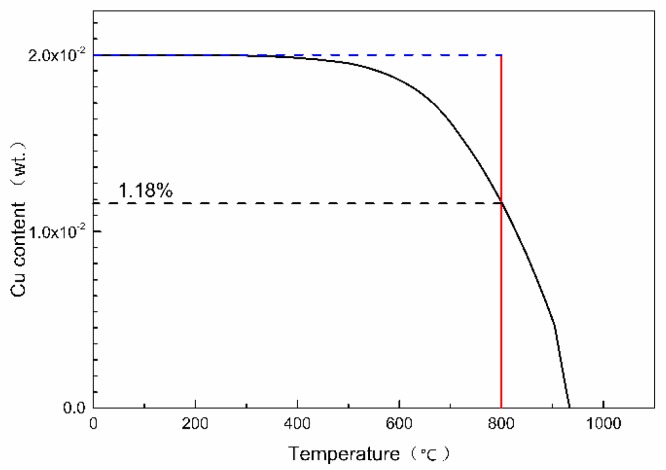
The mass fraction of copper precipitation change with temperature in Fe-17Cr-2Cu alloy.

The optimal antibacterial annealing temperature of Cu-bearing ferrite antibacterial stainless steel is proved to be 800 °C, at which temperature, almost all bacteria are killed. According to the thermodynamic database TCFE7, the mass fraction of copper precipitation in Fe-17Cr-2Cu alloy with temperature can be calculated by Thermo-Calc software, which is shown in Figure 7. What can be seen from the calculation result is that 1.18 wt.% of Cu can be precipitated as copper precipitation when the annealing temperature is 800 °C. At the same time, 0.82 wt.% Cu is dissolved in the matrix as a solid solution. When the samples cool down to room temperature, the amount of copper dissolved in the matrix is still supersaturated. In addition, antibacterial annealing time is not long enough to reach a thermodynamic equilibrium. The Cu content in the matrix is more than 0.82 wt.%, which is much higher than the solid solubility of copper in the steel matrix. Therefore, when the tested steel is annealed at a low temperature, the supersaturated Cu element in the matrix can be nucleated and precipitated. Therefore, from the thermodynamic point of view, the age hardening effect of copper in antibacterial stainless steel can be achieved.

### 3.6. Segregation

According to the metallography theory, the defects such as dislocations, subgrain boundaries, grain boundaries, and surfaces in the material can add an additional distortion energy to the system. In order to reduce the distortion energy, the solute atoms will spontaneously diffuse from the original crystal lattice to the defects. Hence, there is always a partial segregation existing in the crystal defect. Based on the statistical mechanics, it is assumed that there is no interaction between the atoms of the segregated element near the crystal defect, and the defect region is considered to be an irregular ideal solid solution according to the equilibrium thermodynamics of segregation, where the relationship of the adsorption of solute atoms on crystal defects is derived [32]:

(1) $$C_{g}\;=\;\frac{C_{0}\exp\frac{\Delta U}{kT}}{1-C_{0}+C_{0}\exp\frac{\Delta U}{kT}}$$

where *C*_g_ and *C*_0_ represent the equilibrium concentration of the solute and the segregation concentration at the defect, respectively. Δ*U* is the free energy of segregation, and *T* is the absolute temperature (K). When *C*_0_ << 1, the above formula can be simplified to:

(2) $$C_{g}\;=\;C_{0}\exp\frac{\Delta U}{kT}$$

Let Δ*G* = N_0_·Δ*U*, then the above formula can be converted into:

(3) $$C_{g}\;=\;C_{0}\exp\frac{\Delta G}{RT}$$

**Figure 8:**
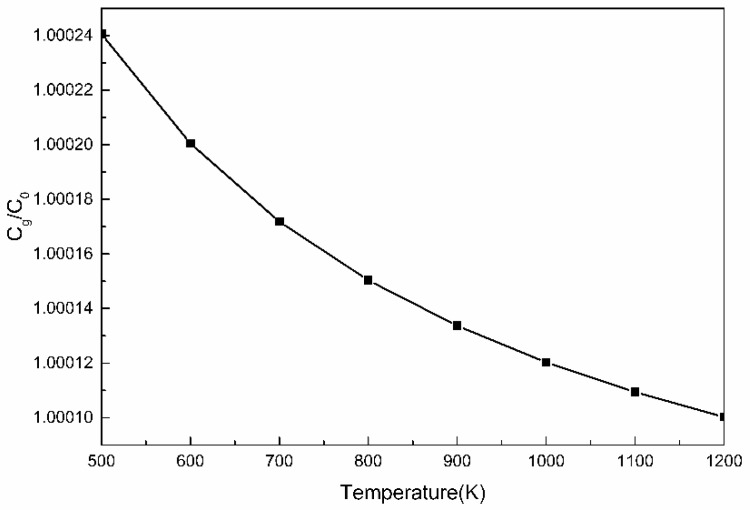
The variation of the enrichment coefficient *C_g_*/*C*_0_ of Cu atoms at crystal defects with temperature.

It can be seen from the above formula, that in addition to the concentration of solute elements, the free energy of segregation is the primary influencing factor that determines the degree of solute segregation. In steel, the upper limit of Δ*G* value of Cu element is 1 J/mol [32]. The relationship between the segregation of Cu element and the temperature calculated according to the Equation (3) is shown in Figure 8.

### 3.7. Nucleating Location

**Figure 9:**
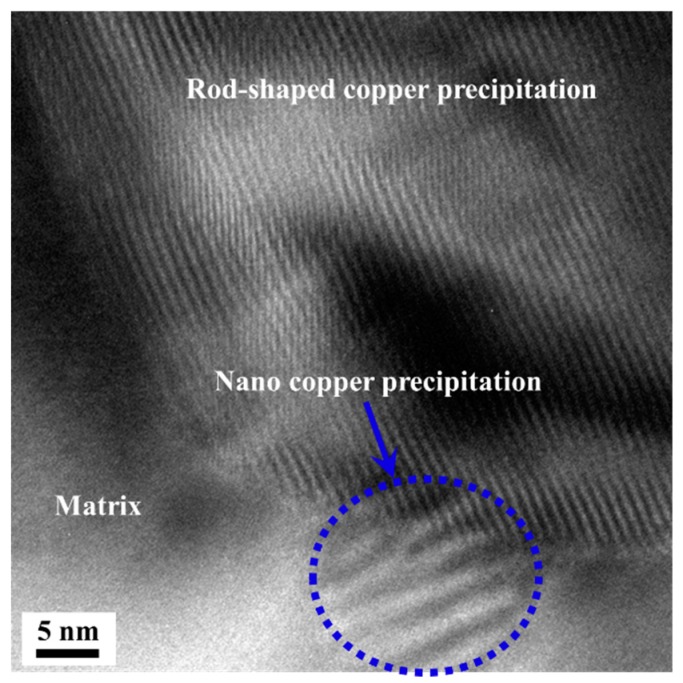
The nano-sized copper precipitation at the interface between rod-shaped copper precipitation and matrix.

It can be seen that a small amount of segregation occurs in the lattice defects of the steel due to the presence of Cu element. This is because the atomic radius of Cu and the atomic radius of Fe and Cr differ by less than 20% [33,34], so the segregation free energy is small. The grade of segregation has a great influence on the nucleating position in this paper. Where there is a high grade of segregation, there is a high probability of nucleation. When the grade of segregation is low, the size of the precipitation is in inverse proportion to the aging temperature, which is because the nuclear driving force is in inverse proportion to the aging temperature; while the diffusion ability of copper atom is proportional to the aging temperature. When aged at low temperature, the nucleation rate is high and the diffusion ability of copper atom is low. Therefore, the nano-scale copper precipitation can be nucleated uniformly after the antibacterial annealing followed by low temperature annealing. Figure 9 shows the nucleation and growth of nano-sized copper precipitation in the matrix. The nucleation of nano-sized copper precipitated at the interface between rod-shaped copper precipitation and matrix can be seen in Figure 9. Twins and Moire’ fringes are found in the particle [35,36].

## 4. Conclusions

This paper investigated the microstructure evolution, variation in mechanical properties and precipitation behavior during the aging process in Cu-bearing antibacterial stainless steel.

1. The microstructure observation shows that nano-sized copper precipitation can be formed during low temperature aging following the antibacterial annealing. Bimodal precipitations are obtained, namely the rod-shaped copper precipitation and nano-sized copper precipitation.
2. Due to the low segregation coefficient of copper, the nano-sized copper precipitations nucleate and grow uniformly in the material. With the increase of aging temperature and aging time, the size of the nano-sized copper precipitation increases. The optimal aging process is aging at 550 °C for one hour.
3. The mechanical properties can be also improved by decreasing the antibacterial annealing time. The best process combination is antibacterial annealing at 800 °C for half an hour followed by aging at 550 °C for one hour. Under this process, the yield strength and tensile strength of the material get the maximum increase. Plasticity and formability is only slightly reduced, and at the same time, the excellent antibacterial property remains.

## Figures and Tables

**Table 1:** Chemical composition of the tested steel (wt.%).

C	Si	Mn	P	S	Cr	Ni	Cu	Fe
0.003	0.19	0.23	0.009	0.001	17.04	0.34	2.07	Balance

**Table 2:** Mechanical properties of the tested steels before and after aging.

Sample	σ_0.2_/MPa	σ_m_/MPa	A/%
No aging	309 ± 3	403 ± 2	27 ± 1.0
Aging	472 ± 3	577 ± 4	22 ± 1.2

Note: σ_m_—the tensile strength, σ_0.2_—the yield strength, A—total elongation.

## References

[1] Xi T., Yang C., Babar Shahzad M., Yang K. (2015). Study of the Processing Map and Hot Deformation Behavior of a Cu-Bearing 317Ln Austenitic Stainless Steel. Mater. Des..

[2] Xiang H., Guo P. (2012). Effects of Antibacterial Aging Treatment on Microstructure and Properties of Copper-Containing Duplex Stainless Steel I. Microstructure and Evolution of Copper-Rich Phase. Acta Metall. Sin..

[3] Xiang H., Gu X. (2012). Effects of Antibacterial Aging Treatment on Microstructure and Properties of Copper-Containing Duplex Stainless Steel Ii. Corrosion Resistance and Antibacterial Properties. Acta Metall. Sin..

[4] Zhang Z.X., Lin G., Xu Z. (2008). Effects of Light Pre-Deformation on Pitting Corrosion Resistance of Copper-Bearing Ferrite Antibacterial Stainless Steel. J. Mater. Process. Technol..

[5] Sun S., Zhao A., Zeng Q., Yin H. (2017). Effect of Continuous Annealing Temperature on Microstructure and Properties of Ultra-Purified Ferritic Stainless Steel. Steel Res. Int..

[6] Ren L., Nan L., Yang K. (2011). Study of Copper Precipitation Behavior in a Cu-Bearing Austenitic Antibacterial Stainless Steel. Mater. Des..

[7] Ren L., Yang K., Guo L., Chai H. (2012). Preliminary Study of Anti-Infective Function of a Copper-Bearing Stainless Steel. Mater. Sci. Eng. C-Mater..

[8] Shypylenko A., Pshyk A.V., Grzeskowiak B., Medjanik K., Peplinska B., Oyoshi K., Pogrebnjak A., Jurga S., Coy E. (2016). Effect of Ion Implantation on the Physical and Mechanical Properties of Ti-Si-N Multifunctional Coatings for Biomedical Applications. Mater. Des..

[9] Ren L., Xu L., Feng J., Zhang Y., Yang K. (2012). In Vitro Study of Role of Trace Amount of Cu Release from Cu-Bearing Stainless Steel Targeting for Reduction of in-Stent Restenosis. J. Mater. Sci. Mater. Med..

[10] Gomes S., Vichery C., Descamps S., Martinez H., Kaur A., Jacobs A., Nedelec J., Renaudin G. (2018). Cu-Doping of Calcium Phosphate Bioceramics: From Mechanism to the Control of Cytotoxicity. Acta Biomater..

[11] Shi M., Chen Z., Farnaghi S., Friis T., Mao X., Xiao Y., Wu C. (2016). Copper-Doped Mesoporous Silica Nanospheres, a Promising Immunomodulatory Agent for Inducing Osteogenesis. Acta Biomater..

[12] Sun S., Yin F., Liu Y., Zhang W., Zhao A., Han Q. (2018). Deformation-Induced Dissolution of Copper Precipitation in 1.5Wt%Cu-Bearing Antibacterial Fe-17Wt%Cr Alloy during Plastic Deformation Process. Mater. Des..

[13] Bahmani-Oskooee M., Hossein Nedjad S., Samadi A., Kozeschnik E. (2017). Cu-Bearing, Martensitic Stainless Steels for Applications in Biological Environments. Mater. Des..

[14] Wang S., Yang C., Shen M., Yang K. (2014). Effect of Aging on Antibacterial Performance of Cu-Bearing Martensitic Stainless Steel. Mater. Technol..

[15] Dlouhy J., Podany P., Dzugan J. (2019). Strengthening From Cu Addition in 0.2C-(1-2)Mn Steels During Tempering. Materials.

[16] Shi X., Yan W., Wang W., Shan Y., Yang K. (2016). Novel Cu-Bearing High-Strength Pipeline Steels with Excellent Resistance to Hydrogen-Induced Cracking. Mater. Des..

[17] Holzer I., Kozeschnik E. (2010). Computer Simulation of the Yield Strength Evolution in Cu-Precipitation Strengthened Ferritic Steel. Mater. Sci. Eng. A.

[18] Rana R., Bleck W., Singh S.B., Mohanty O.N. (2007). Development of High Strength Interstitial Free Steel by Copper Precipitation Hardening. Mater. Lett..

[19] Zhou W.H., Guo H., Xie Z.J., Shang C.J., Misra R.D.K. (2014). Copper Precipitation and its Impact on Mechanical Properties in a Low Carbon Microalloyed Steel Processed by a Three-Step Heat Treatment. Mater. Des..

[20] Guo H., Cheng J., Yang S., He X. (2013). Influence of Combined Cu and Nb Addition on the Quenched Microstructure and Precipitation during Tempering in Ultra-Low Carbon Steels. J. Alloys Compd..

[21] Misra R.D.K., Jia Z., O’Malley R., Jansto S.J. (2011). Precipitation Behavior during Thin Slab Thermomechanical Processing and Isothermal Aging of Copper-Bearing Niobium-Microalloyed High Strength Structural Steels: The Effect on Mechanical Properties. Mater. Sci. Eng. A-Struct..

[22] Han G., Xie Z.J., Xiong L., Shang C.J., Misra R.D.K. (2017). Evolution of Nano-Size Precipitation and Mechanical Properties in a High Strength-Ductility Low Alloy Steel through Intercritical Treatment. Mater. Sci. Eng. A.

[23] Kang J., Heo Y., Kim H., Suh D., Son D., Lee D.H., Lee T. (2014). Effect of Copper Addition on the Characteristics of High-Carbon and High-Chromium Steels. Mater. Sci. Eng. A.

[24] Karunaratne M.S.A., Yan S., Thomson R.C., Coghlan L., Higginson R.L. (2019). Modelling carburisation in 9Cr-1Mo ferritic steel tube substrates in experimental CO_2_ atmospheres. Corros. Sci..

[25] Kim M.T., Park T.M., Baik K.H., Won S.C., Pyuck-Pa C., Jeongho H. (2019). Crucial microstructural feature to determine the impact toughness of intercritically annealed medium-Mn steel with triplex-phase microstructure. Acta Mater..

[26] Andersson J.O., Helander T., Höglund L., Shi P., Sundman B. (2002). Thermo-Calc & DICTRA, computational tools for materials science. Calphad.

[27] Yin H. (2016). Copper Precipitation Mechanism and Performance Control of Ferritic Antibacterial Stainless Steel. Ph.D. Thesis.

[28] Yin H., Zhai A., Zhao Z., Zhou K., Pei W., Yan Y. (2015). Effects of annealing time on microstructure and properties of ultra purified ferritic stainless steel containing copper. Heat Treat. Met..

[29] Lin G., Shen J., Wang R. (2011). Effect of Cu on the property of ferrite antibacterial stainless steel. J. Funct. Mater..

[30] Zou D., Zhang W., Zhang S., Bai J., Lin Q., Han Y. (2005). Effect of antibacterial treatment on microstructure and properties of copper-bearing ferritic antibacterial stainless steel. Foundry Technol..

[31] Sun S., Zhao A., Ding R., Li X. (2018). Effect of Heat Treatment on Microstructure and Mechanical Properties of Quenching and Partitioning Steel. Acta Metall. Sin. Engl. Lett..

[32] Yong Q. (2006). Secondary Phase in Steels.

[33] Pyykkö P. (2014). Additive Covalent Radii for Single-, Double-, and Triple-Bonded Molecules and Tetrahedrally Bonded Crystals: A Summary. J. Phys. Chem. A.

[34] Tsuchiyama T., Yamamoto S., Hata S., Murayama M., Morooka S., Akama D., Takaki S. (2016). Plastic Deformation and Dissolution of Ε-Cu Particles by Cold Rolling in an Over-Aged Particle Dispersion Strengthening Fe-2Mass%Cu Alloy. Acta Mater..

[35] Sun M., Zhang W., Liu Z., Wang G. (2017). Direct Observations on the Crystal Structure Evolution of Nano Cu-Precipitates in an Extremely Low Carbon Steel. Mater. Lett..

[36] Wang Z., Li H., Shen Q., Liu W., Wang Z. (2018). Nano-Precipitates Evolution and their Effects on Mechanical Properties of 17-4 Precipitation-Hardening Stainless Steel. Acta Mater..

